# Effect of oxygen limitation on the enrichment of bacteria degrading either benzene or toluene and the identification of *Malikia spinosa* (Comamonadaceae) as prominent aerobic benzene-, toluene-, and ethylbenzene-degrading bacterium: enrichment, isolation and whole-genome analysis

**DOI:** 10.1007/s11356-020-09277-z

**Published:** 2020-05-30

**Authors:** Fruzsina Révész, Milán Farkas, Balázs Kriszt, Sándor Szoboszlay, Tibor Benedek, András Táncsics

**Affiliations:** 1 Regional University Center of Excellence in Environmental Industry, Szent István University, Gödöllő, Hungary; 2 Department of Environmental Protection and Safety, Szent István University, Gödöllő, Hungary

**Keywords:** *Malikia spinosa*, BTEX degradation, Bioremediation, Petroleum hydrocarbons, Groundwater

## Abstract

The primary aims of this present study were to evaluate the effect of oxygen limitation on the bacterial community structure of enrichment cultures degrading either benzene or toluene and to clarify the role of *Malikia*-related bacteria in the aerobic degradation of BTEX compounds. Accordingly, parallel aerobic and microaerobic enrichment cultures were set up and the bacterial communities were investigated through cultivation and 16S rDNA Illumina amplicon sequencing. In the aerobic benzene-degrading enrichment cultures, the overwhelming dominance of *Malikia spinosa* was observed and it was abundant in the aerobic toluene-degrading enrichment cultures as well. Successful isolation of a *Malikia spinosa* strain shed light on the fact that this bacterium harbours a catechol 2,3-dioxygenase (C23O) gene encoding a subfamily I.2.C-type extradiol dioxygenase and it is able to degrade benzene, toluene and ethylbenzene under clear aerobic conditions. While quick degradation of the aromatic substrates was observable in the case of the aerobic enrichments, no significant benzene degradation, and the slow degradation of toluene was observed in the microaerobic enrichments. Despite harbouring a subfamily I.2.C-type C23O gene, *Malikia spinosa* was not found in the microaerobic enrichments; instead, members of the *Pseudomonas veronii*/*extremaustralis* lineage dominated these communities. Whole-genome analysis of *M. spinosa* strain AB6 revealed that the C23O gene was part of a phenol-degrading gene cluster, which was acquired by the strain through a horizontal gene transfer event. Results of the present study revealed that bacteria, which encode subfamily I.2.C-type extradiol dioxygenase enzyme, will not be automatically able to degrade monoaromatic hydrocarbons under microaerobic conditions.

**Electronic supplementary material:**

The online version of this article (10.1007/s11356-020-09277-z) contains supplementary material, which is available to authorized users.

## Introduction

Monoaromatic pollutants such as benzene, toluene, ethylbenzene and isomers of xylene (BTEX compounds) are volatile organic hydrocarbons. BTEX compounds are produced and used during the processing of petroleum products and widely used in industry, e.g. in the manufacture of paints, varnishes, insecticides, pharmaceuticals and solvents. Besides extraction, usage, storage and transport, accidentally they are often released into the environment, causing a serious threat to soil and groundwater ecosystems. They are relatively soluble in water, therefore tended to spread quickly in the direction of groundwater movement. Each BTEX compound can cause neurological disturbances; in addition, benzene can cause hematological effects including aplastic anemia and acute myelogenous leukemia in humans (Fenga et al. 2016).

In bioremediation procedures, bacteria are used to eliminate xenobiotics. Some microorganisms are able to use a wide range of petroleum derivatives as a source of carbon and energy for their metabolic processes. It is well known that aromatic hydrocarbons, such as the BTEX compounds, decompose most rapidly and completely under aerobic conditions (El-Naas et al. 2014). Aerobic BTEX degraders use mono- and dioxygenases for the hydroxylation and the subsequent cleavage of the aromatic ring. Consequently, these enzymes require molecular oxygen as a co-substrate. However, in polluted subsurface environments, the availability of dissolved oxygen is often limited. In these environments, a specific group of extradiol dioxygenases (subfamily I.2.C), adapted to low oxygen concentrations (Kukor and Olsen 1996), can have a key role in the catalysis of the ring cleavage reaction. Recently, evidence was found for the ring-cleavage activity of this I.2.C-type extradiol dioxygenase (catechol 2,3-dioxygenase, C23O) in the case of *Zoogloea oleivorans* degrading toluene under microaerobic conditions (Táncsics et al. 2020). On the other hand, not all of the I.2.C C23O genotypes can be linked to the ability of microaerobic BTEX degradation, as it was shown in our previous enrichment study (Benedek et al. 2018). We have revealed that different subfamily I.2.C-type C23O genes and different members of the Betaproteobacteriales were dominant under aerobic versus microaerobic conditions in BTEX-degrading enrichment cultures (Benedek et al. 2018). In the aerobic BTEX-degrading enrichments, members of the genus *Malikia* were dominant, while members of the genus *Acidovorax* were the most abundant under microaerobic conditions. Unfortunately, isolation of representatives of these dominant genera was not succeeded; therefore, the dominant subfamily I.2.C-type C23O genotypes were linked to them only theoretically (Benedek et al. 2018).

The main aim of the present study was to re-enrich and isolate *Malikia-*related bacteria, to confirm our presumption that they can harbour subfamily I.2.C-type C23O gene, and that they can play role in the degradation of certain BTEX compounds, but solely under clear aerobic conditions. For this, aerobic and microaerobic enrichment cultures were set up by using biofilm material originated from the same site as in the case of our previous enrichment (Benedek et al. 2018).

## Materials and methods

### Enrichment setup and process measurements

To reach the goals of the present study, aerobic and microaerobic enrichments were set up in triplicate. For the inoculation of the microcosms, biofilm sample was obtained from a petroleum hydrocarbon–contaminated site, located in central Hungary. Previous studies have shown the diverse occurrence of hydrocarbon-degrading bacteria in this biofilm material (Benedek et al. 2016, 2018). To assemble the enrichment microcosms, 100-mL serum bottles were used, sealed with butyl-rubber septa and aluminium crimp seals. Fifty milliliters of mineral salt (MS) medium (Fahy et al. 2006) was used for each enrichment culture supplemented with vitamins (1 mg/L thiamine, 15 μg/L biotin and 20 μg/L vitamin B_12_), and then amended with benzene or toluene as sole carbon and energy source at a concentration of 1 mM.

Before inoculation of the microaerobic enrichments, microcosms were sparged with N_2_/CO_2_ (80:20, v/v) for 10 min. The desired concentration of dissolved oxygen was then adjusted by injection of sterile air (0.2 μm pore size filtered) through the butyl-rubber septa. Initial enrichment cultures were inoculated with 1 g (wet weight) of biofilm sample. Changes in dissolved oxygen concentration in the bottles were monitored non-invasively by Fibox 3 trace v3 fibre optic oxygen meter with PSt3 sensor spots (PreSens). To maintain aerobic and microaerobic conditions, oxygen was continuously replenished in the enrichments. Microcosms were incubated in a shaking thermostat (28 °C, 150 rpm) for 1 week, and then 5 mL of each enrichment was inoculated into fresh MS medium. The transfers were carried out for five consecutive weeks.

Concentrations of benzene and toluene were determined in the case of the 5th week enrichments in every 24 h by headspace analysis on an ISQ Single Quadrupole gas chromatography–mass spectrometer (GC–MS) (Thermo Fischer Scientific) via a SLB-5ms fused silica capillary column (Supelco Analytical). The oven temperature was set to 40 °C for 3 min, then ramped at a rate of 20 °C min^−1^ to 190 °C, and held for 1 min. The mass spectrometer was operated at 250 °C in full scan mode. Triplicates of uninoculated bottles with 50 mL of MS medium containing either 1 mM benzene or toluene served as negative controls for the GC–MS measurements.

### DNA isolation and T-RFLP fingerprinting

After five consecutive transfers, the microbial biomass was harvested from 50 mL enrichments by centrifugation at 2360*g* at 4 °C for 10 min using a Rotanta 460 R (Hettich) and DNA was isolated from the pellets by using the DNeasy UltraClean Microbial Kit (Qiagen) according to the instructions of the manufacturer. To isolate DNA from the biofilm inoculum, the NucleoSpin Soil Kit (Macherey-Nagel) was used by following the instructions of the manufacturer. For 16S rDNA–based T-RFLP community fingerprinting, the VIC-labelled PCR amplicons were generated by using 27f-VIC (5′-AGA GTT TGA TCM TGG CTC AG-3′) and 1492r primers (5′-TACGGYTACCTTGTTACGACTT-3′) as described earlier (Benedek et al. 2016). The PCR reaction mixture (final volume of 50 μL) included: 5 μL of 10x DreamTaq Buffer (Thermo Fischer Scientific), 0.2 mM of each of the four dNTP, 0.3 μM of each primer, 0.25 μL of a 5-U μL^−1^ DreamTaq DNA Polymerase solution (Thermo Fisher Scientific), 20 ng template DNA and autoclaved MilliQ water up to 50 μL. All amplifications were performed in a ProFlex PCR System (Life Technologies). All amplification products were checked by electrophoresis on 1% agarose gels stained with ethidium bromide.

The obtained VIC-labelled 16S rDNA PCR products were digested with 1 U *Rsa*I (GT↓AC) restriction enzyme (Thermo Fischer Scientific) for 1.5 h at 37 °C. The generated fluorescently labelled terminal restriction fragments (T-RFs) were purified by ethanol precipitation method. After ethanol precipitation, fragments were separated on a Model 3130 Genetic Analyzer (Applied Biosystems), while primary evaluation of electropherograms was performed using GeneMapper 4.0 software (Applied Biosystems). T-RFLP data were handled as described earlier (Farkas et al. 2017). Cluster analysis (Bray-Curtis method) of the T-RFLP electropherograms was performed by using the PAST 3.26 software package (Hammer et al. 2001).

### 16S rDNA amplicon sequencing and data handling

16S rDNA amplicon sequencing was carried out from the initial biofilm sample as well as from one representative of the triplicate fifth week enrichment cultures. The variable V3 and V4 regions of the 16S rRNA gene were amplified by using the PCR primers suggested by Klindworth et al. (2013). KAPA HiFi HotStart Ready Mix-et (KAPA Biosystems) was used for PCR according to the 16S metagenomics sequencing library preparation guide of Illumina. Paired-end fragment reads were generated on an Illumina MiSeq sequencer using MiSeq Reagent Kit v3 (600-cycle) by SeqOmics Biotechnology Ltd. (Mórahalom, Hungary). Read numbers were the following: 33,778 for enrichment AB1; 29,721 for enrichment AT2,; 33,056 for enrichment MB1; 36,823 for enrichment MT3 and 33,820 for initial biofilm sample (BUT18). Primary data analysis (base calling) was carried out with Bbcl2fastq^ software (v2.17.1.14, Illumina). Reads were quality and length trimmed in CLC Genomics Workbench Tool 9.5.1 using an error probability of 0.05 (Q13) and a minimum length of 50 nucleotides as a threshold. Trimmed sequences were processed using mothur v1.41.1 (Schloss et al. 2009) as recommended by the MiSeq SOP page (http://www.mothur.org/wiki/MiSeq_SOP) (Kozich et al. 2013). Sequences were assorted based on the alignment using SILVA 132 SSURef NR99 database (Quast et al. 2013). Chimera detection was carried out with mothur’s uchime command (Edgar et al. 2011), and ‘split.abund’ command was also used to remove singleton reads according to Kunin et al. (2010). Taxonomic assignments were made against SILVA release 123 applying a minimum bootstrap confidence score of 80%. Operational taxonomic units (OTUs) were assigned at 97% similarity threshold level as suggested by Tindall et al. (2010) for prokaryotic species delineation. Sequence reads were deposited in NCBI SRA under BioProject ID PRJNA433949 (Inoculum: SRX7512837, aerobic benzene-degrading enrichment culture AB1: SRX7512838, aerobic toluene-degrading enrichment culture AT2: SRX7512839, microaerobic benzene-degrading enrichment culture MB1: SRX7512840 and microaerobic toluene-degrading enrichment culture MT3: SRX7512841).

### Cloning of I.2.C C23O amplicons, Sanger-sequencing and phylogenetic analysis

Subfamily I.2.C-type *C23O* genes were PCR amplified by using the primers XYLE3f (5′-TGY TGG GAY GAR TGG GAY AA-3′) and XYLE3r (5′-TCA SGT RTA SAC ITC SGT RAA-3′) and were cloned and sequenced as described earlier (Táncsics et al. 2012; Táncsics et al. 2013). The *C23O* sequences of each clone library were manually grouped into operational protein units (OPUs) by applying a cutoff value of 0.03. Terminal restriction fragments (T-RFs) predicted in silico for representative clones of each of the OPUs were verified in vitro. The neighbour-joining phylogenetic tree was reconstructed based on the deduced amino-acid sequences by using MEGA ver. 7.0 (Kumar et al. 2016). For tree reconstruction the Jones-Taylor-Thornton model was used and gaps were treated by complete deletion; the number of bootstrap replications was set to 1000, while the substitution rates were set to be the same among the sites and lineages. Representative clones of each OPU were deposited with GenBank and can be found under the accession numbers MN823973-MN823982.

### Isolation and identification of bacterial strains from the enrichments

To gain bacterial isolates from the enrichments, serially diluted samples were spread on the surface of R2A agar plates (proteose peptone 0.5 g, casamino acids 0.5 g, yeast extract 0.5 g, dextrose 0.5 g, soluble starch 0.5 g, dipotassium phosphate 0.3 g, MgSO4 7H_2_O 0.05 g, sodium pyruvate 0.3 g, agar 15 g, pH 7 ± 0.2). Chemicals were obtained from Sigma-Aldrich, Germany. After 1 week of incubation (28 °C), colonies with different morphologies were purified by streak plating technique and maintained on R2A agar slants at 28 °C. DNA isolation from the isolates was performed by using the UltraClean Microbial DNA Kit (Qiagen) according to the instructions of the manufacturer. The 16S rRNA genes of the isolates were amplified by using the bacterial PCR primers 27f and 1492r, while XYLE3f/XYLE3r primer set was used to amplify the subfamily I.2.C-type C23O genes as described above. Sequencing of the 16S rDNA and C23O amplicons was performed according to Benedek et al. (2018). The 16S rRNA and C23O gene sequences of the isolates were deposited with GenBank under the accession numbers MN449442-MN449463 and MN481621-MN481623, respectively. Transmission electron microscopic observations were performed by applying negative staining (Szoboszlay et al. 2008) and the shadow-casting technique (Ohad et al. 1963), respectively.

### BTEX-degradation analysis of the isolates

The ability of the isolates to degrade individual BTEX compounds under aerobic conditions was investigated by GC–MS analysis. Measurements were carried out in triplicates of 100 mL crimped serum bottles containing 50 mL of MS medium, amended with an individual BTEX compound at a concentration of 5 mg/L. Enrichments were inoculated with 100 μL bacterial suspensions (OD600 0.5) and incubated in a rotary shaker at 28 °C and 150 rpm. Uninoculated bottles served as negative controls. Concentrations of BTEX compounds were determined in every 24-h interval by headspace GC–MS analysis as detailed above.

### Whole-genome sequencing and comparative genomics analysis

Whole-genome sequencing was performed as described previously by SeqOmics Biotechnology Ltd. (Mórahalom, Hungary) (Borsodi et al. 2019), briefly: Nextera Mate Pair Sample Preparation Kit (Illumina) was used to generate mate-paired libraries according to the manufacturer’s protocol for gel-plus version with slight modifications. Thirteen microliters of Mate Pair Tagment Enzyme was used to produce a robust smear within the 7–11 kbp region. The 7–11 kbp DNA fraction was excised from the gel using the Zymoclean Large Fragment DNA Recovery kit (Zymo Research), and the circularized DNA was sheared using Covaris S2. All quality measurements were performed on a TapeStation 2200 instrument (Agilent). Final libraries were quantified using Qubit (ThermoFisherScientific) and sequenced on an Illumina MiSeq instrument using MiSeq Reagent Kit v2 (500 cycles) sequencing chemistry. De novo assembly and scaffolding was performed with CLC Genomics Workbench Tool v11 (Qiagen). Automatic annotation of the genome was performed by the NCBI Prokaryotic Genomes Automatic Annotation Pipeline (PGAP) v4.5 (Tatusova et al. 2016). Annotation was also performed by using the RAST server (through the RASTtk scheme) and the SEED database (Aziz et al. 2008; Overbeek et al. 2014; Brettin et al. 2015). Graphical visualization of gene clusters was performed by using SnapGene v4.3.4. To perform digital DNA-DNA hybridization analysis, the online available genome-to-genome distance calculator (GGDC, version 2.1) was used (Meier-Kolthoff et al. 2013). To calculate orthologous average nucleotide identity value (OrthoANI), the OAT software was used (Lee et al. 2016). OrthoVenn2 software (Xu et al. 2019) was used to perform comparative genomics analysis.

## Results and discussion

### Microbial community compositions as revealed by 16S rDNA amplicon sequencing

For the inoculation of the enrichment cultures, a biofilm material was used, which was taken from a groundwater well of an extensively studied Hungarian petroleum hydrocarbon–contaminated site (Benedek et al. 2016). The sampled biofilm continuously developed on the stainless steel surface of a submersible pump, which was part of a pump-and-treat system operating at the contaminated site. In previous enrichment studies, this continuously emerging biofilm was successfully used to study the biodegradation of petroleum hydrocarbons under microaerobic conditions (Benedek et al. 2018; Révész et al. 2020). Members of the class Gammaproteobacteria overwhelmingly dominated the bacterial community of the newly sampled biofilm material (47% of 16S rDNA sequence reads) (Online Resource 1, Fig. S1). Moreover, mainly genera of the Betaproteobacteriales were abundant, such as members of the *Sulfuritaela* (9.7%), *Azoarcus* (6.5%), *Zoogloea* (4.7%), *Simplicispira* (3%), *Thauera* (2.4%), *Denitratisoma* (1%), *Leptothrix* (0.9%), *Acidovorax* (0.8%) and *Malikia* (0.7%). Many of these genera contain well-known aromatic hydrocarbon degraders (e.g. *Azoarcus*, *Zoogloea*, *Thauera* and *Acidovorax*) (Prince et al. 2018), or frequently found in petroleum hydrocarbon–contaminated subsurface environments (e.g. *Sulfuritalea*, *Simplicispira* and *Denitratisoma*) (Sperfeld et al. 2018). Due to this enormous diversity of potentially petroleum hydrocarbon–degrading bacteria, the biofilm material was an excellent inoculum for the benzene- and toluene-degrading enrichments. Most importantly, *Malikia*-related bacteria were detectable in this biofilm community.

**Fig. 1:**
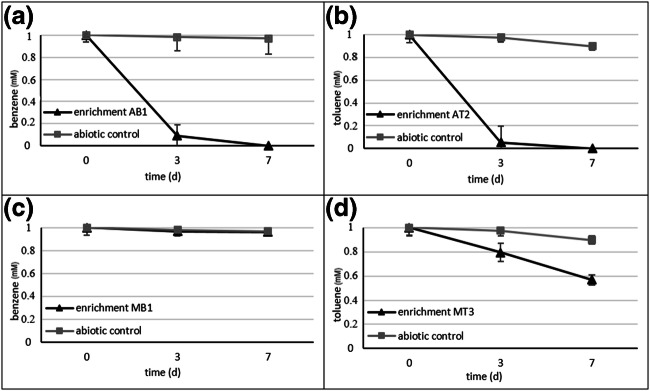
Degradation process of aromatic hydrocarbons in the aerobic enrichments containing **a** benzene, or **b** toluene and in the microaerobic enrichments containing **c** benzene, or **d** toluene. Benzene and toluene concentrations were measured by GC–MS analysis at the 5th week of enrichment as described in the main text. Means of triplicate enrichments are given (with standard error)

By using the biofilm inoculum, aerobic (7–8 mg/L dissolved oxygen) and microaerobic enrichments (0.5 mg/L dissolved oxygen) were set up in triplicates with either benzene or toluene as a sole source of carbon and energy. Enrichments were then transferred weekly for consecutive 5 weeks. At the fifth week, aromatic hydrocarbon degradation processes in the enrichments reflected that highly efficient degrading communities evolved in the aerobic benzene- and toluene-degrading enrichments, respectively. In these enrichments, the carbon source was almost depleted by the third day of incubation, and no aromatic hydrocarbon was detectable by the seventh day (Fig. 1 a and b). On the other hand, under microaerobic conditions, the level of aromatic hydrocarbon degradation was significantly lower. Notable degradation was observable only in the toluene-containing enrichments, in which ~ 40% of toluene was degraded by the seventh day of incubation (Fig. 1d), while in the benzene-containing enrichments no significant degradation was observed (Fig. 1c). After the fifth week of enrichment, the bacterial community compositions were first investigated by using 16S rDNA–based T-RFLP. The Bray-Curtis similarity–based dendrogram of the T-RFLP profiles showed that the bacterial communities of the aerobic and microaerobic enrichments were entirely different (Online Resource 1, Fig. S2). The aerobic enrichments degrading either benzene or toluene belonged to one clear group of the dendrogram, but it was also observable that different subgroups of the dendrogram contained the benzene- and the toluene-degrading enrichments. On the other hand, the microaerobic enrichments separated into two distinct groups, depending on the type of aromatic hydrocarbon (i.e. benzene or toluene). Furthermore, it was also noticeable that the triplicate enrichments had mostly similar community compositions. Accordingly, one representative of each triplicate enrichment was chosen for detailed community analysis.

**Fig. 2:**
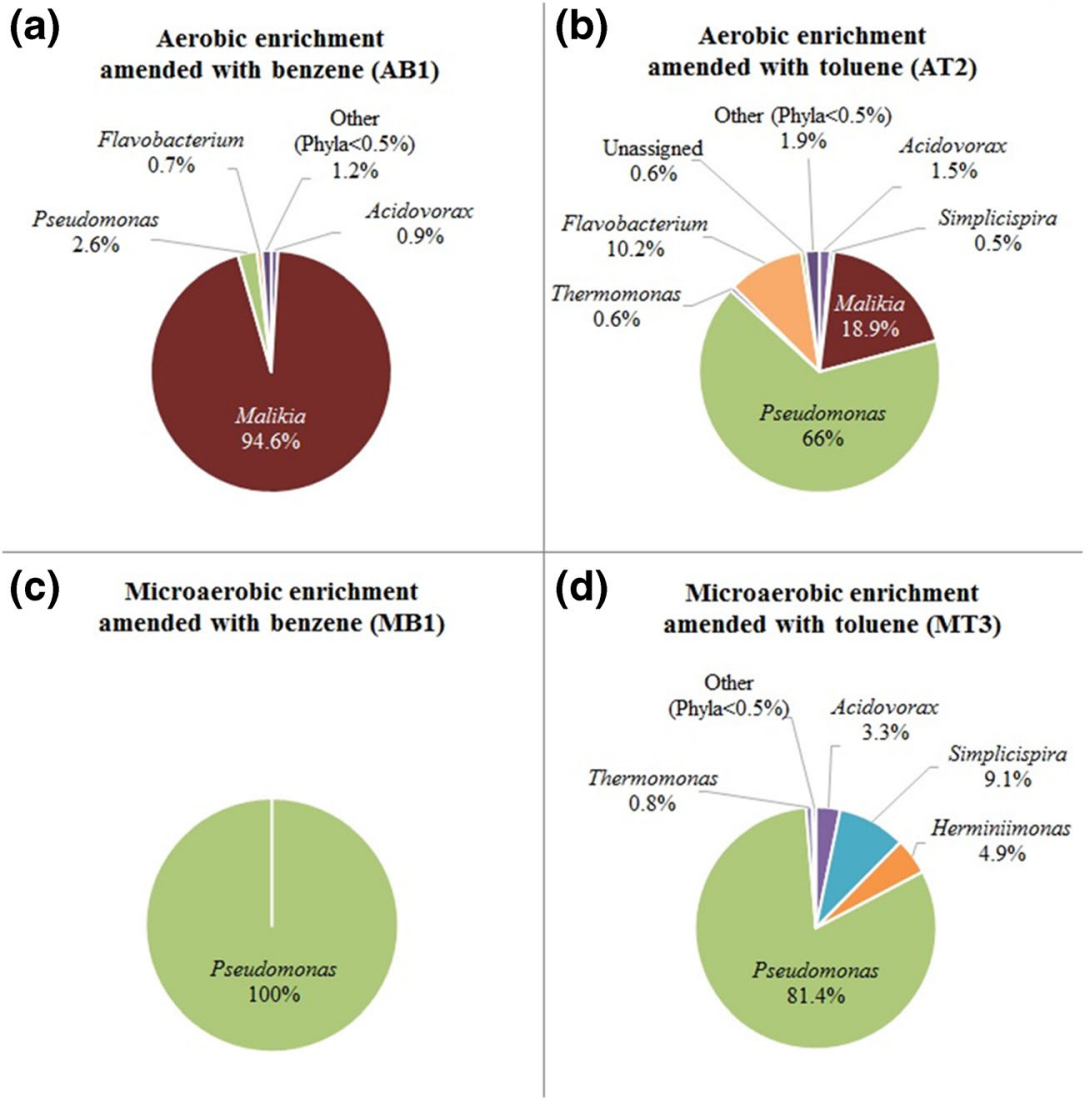
Genus level bacterial community structure of enrichments AB1, AT2, MB1 and MT3 as revealed by Illumina paired-end 16S rDNA amplicon sequencing. All taxa contributing more than 0.5% abundance were depicted

The bacterial community of the aerobic benzene-degrading enrichment (AB1) was dominated by a single genus, since more than 94% of 16S rDNA sequence reads could be linked to the genus *Malikia* (Betaproteobacteriales, Comamonadaceae) (Fig. 2a). Besides, members of the genera *Pseudomonas*, *Acidovorax* and *Flavobacterium* were detectable with notable abundance (> 0.5%). At the time of writing, the genus *Malikia* contained two species. The type species of the genus is *M. granosa*, which was described as a polyhydroxyalkanoate- and polyphosphate-accumulating bacterium, isolated from wastewater activated sludge (Spring et al. 2005). The other member of the genus was isolated from river water and described as *Pseudomonas spinosa* (Leifson 1962), and later reclassified as *M. spinosa* by Spring et al. (2005). Although the occurrence of the genus *Malikia* in BTEX-contaminated subsurface environments has already been reported by some studies (Aburto and Ball 2009; Táncsics et al. 2010) though their exact role remained hidden. The closest relatives of *Malikia* species can be found in the genus *Hydrogenophaga*, which contains excellent BTEX degraders (Fahy et al. 2008; Jechalke et al. 2013). Nevertheless, *Malikia* isolates with aromatic hydrocarbon–degrading ability have not been reported yet in the literature. Our previous enrichment study enabled to speculate that these bacteria could be active aromatic hydrocarbon degraders (Benedek et al. 2018).

The bacterial community of the aerobic toluene-degrading enrichment (AT2) was dominated by members of the genus *Pseudomonas* (66%), followed by *Malikia* (18.9%), *Flavobacterium* (10.2%) and *Acidovorax* (1.5%) (Fig. 2b). The dominant role of *Pseudomonas*-related bacteria in toluene degradation under fully aerobic conditions is well known. Consequently, these bacteria are widely investigated as model organisms for the study of aerobic toluene degradation. The high abundance of sequence reads affiliated with the genus *Malikia* enabled to assume that these bacteria also played role in the aerobic degradation of toluene. This ability has never been linked before to these bacteria. On the other hand, members of the genera *Flavobacterium* and *Acidovorax* are frequently observed in petroleum hydrocarbon–contaminated environments (Kaplan and Kitts 2004; Aburto et al. 2009; Singleton et al. 2018).

The bacterial community of the microaerobic, benzene-containing enrichment (MB1) was considerably simple, since all of the 16S rDNA sequence reads could be affiliated with the genus *Pseudomonas* (Fig. 2c). Nevertheless, no significant benzene degradation was observed in this enrichment culture. It is highly assumable that these *Pseudomonas* bacteria were feeding on cell debris or secondary metabolites of other bacteria, which were present in the enrichment only in trace amounts. Contrarily, the bacterial community of the microaerobic, toluene-degrading enrichment (MT3) had a bit larger diversity (Fig. 2d). Although still members of the genus *Pseudomonas* dominated the community (81%), members of the genera *Simplicispira* (9.1%), *Herminiimonas* (4.9%) and *Acidovorax* (3.3%) were also detected with notable abundance. Members of the genus *Herminiimonas* probably took part in the degradation of toluene during periods when oxygen was depleted from the enrichments and anaerobic conditions prevailed. This can be assumed based on the study of Kim et al. (2014) which reported on an uncultivated *Herminiimonas*-related bacterium capable of degrading toluene under nitrate-reducing conditions. The metagenome-assembled genome of this bacterium contained genes encoding the benzylsuccinate synthase, which is the key enzyme of anaerobic toluene degradation and known to be highly oxygen sensitive (Leuthner et al. 1998). Members of genera *Simplicispira* and *Acidovorax* are frequently observed in oxygen-limited, petroleum hydrocarbon–degrading enrichment cultures (Keller et al. 2018; Révész et al. 2020); therefore, their role in the degradation can be also assumed.

### Diversity and phylogenetic affiliation of subfamily I.2.C-type C23O gene clones

**Fig. 3:**
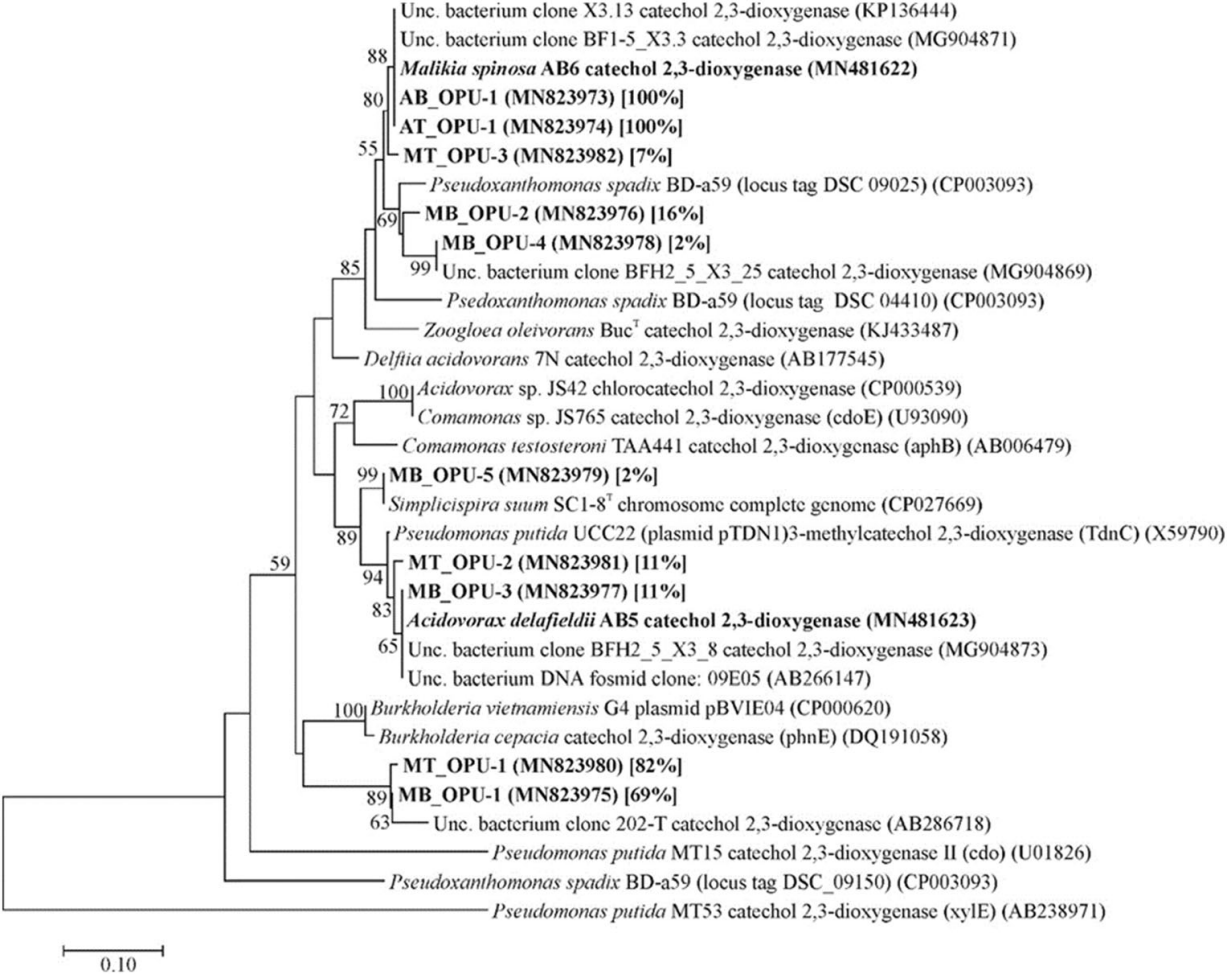
Neighbour-joining tree showing the phylogenetic position of subfamily I.2.C-type C23O amino acid sequences retrieved from enrichments and bacterial isolates. Bootstrap values from 1000 re-samplings are indicated at the branches (only values > 50% are shown). OPUs were determined using a distance cutoff of 0.03 (97% sequence similarity). The tree was rooted with subfamily I.2.A-type catechol 2,3-dioxygenase (xylE) amino acid sequence coded on plasmid pWW53 of *Pseudomonas putida* MT53

In the case of those enrichments, which were analysed by Illumina 16S rDNA amplicon sequencing, the diversity of subfamily I.2.C-type C23O genes was also recovered. In the aerobic benzene-degrading enrichment culture (AB1), all of the subfamily I.2.C-type C23O gene sequences belonged to a single OPU (AB_OPU-1). Moreover, this C23O genotype was identical with that of was found earlier by us in the case of an aerobic BTEX-degrading enrichment culture (Fig. 3) and was putatively linked to the genus *Malikia* (Benedek et al. 2018). Surprisingly, this was the only detectable subfamily I.2.C-type C23O genotype in the aerobic toluene-degrading enrichment culture (AT2) as well (AT_OPU-1). Although no significant benzene degradation was found in the microaerobic enrichments, the largest C23O diversity was detectable in the case of enrichment MB1, since the C23O sequences could be grouped into five OPUs. This observation is in contradiction with the considerably low bacterial diversity that we have recovered in the case of this enrichment. However, this result suggests that beside *Pseudomonas*-related bacteria, members of other genera were also present in the community of enrichment MB1, although most probably in trace amounts only. Most of the C23O sequences in this enrichment belonged to MB_OPU-1 (69% of clone sequences), which was most closely related to a C23O of an unknown bacterium. Sequences of the MB_OPU-2 and MB_OPU-4 were most closely related to a C23O sequence, which was retrieved earlier by us from a hypoxic BTEX-degrading enrichment culture (Benedek et al. 2018). Similarly, sequences of MB_OPU-3 showed high degree of similarity to a C23O genotype, which was the dominant one in the aforementioned hypoxic BTEX-degrading enrichment culture. Previously, we have putatively linked this subfamily I.2.C C23O genotype to members of the genus *Acidovorax* (Benedek et al. 2018). Finally, the MB_OPU-5 contained a single C23O sequence, which was almost identical with the C23O gene sequence of *Simplicispira suum* strain SC1-8. Regarding C23O clone sequences of the microaerobic toluene-degrading enrichment culture MT3, they could be grouped into three OPUs. The dominant C23O sequences belonged to MT_OPU-1 and were identical with sequences of MB_OPU-1 of the microaerobic enrichment MB1. Consequently, the same subfamily I.2.C-type C23O genotype was dominant in both types of microaerobic enrichments. Sequences belonging to MT_OPU-2 showed high degree of similarity with sequences of MB_OPU-3, while MT_OPU-3 contained sequences, which were highly similar to sequences of OPUs of the aerobic enrichments. Overall, it can be concluded that mainly the availability of oxygen shaped the diversity of subfamily I.2.C-type C23O genes in the enrichments, rather than the type of aromatic hydrocarbon (i.e. benzene versus toluene).

### Isolates

**Fig. 4:**
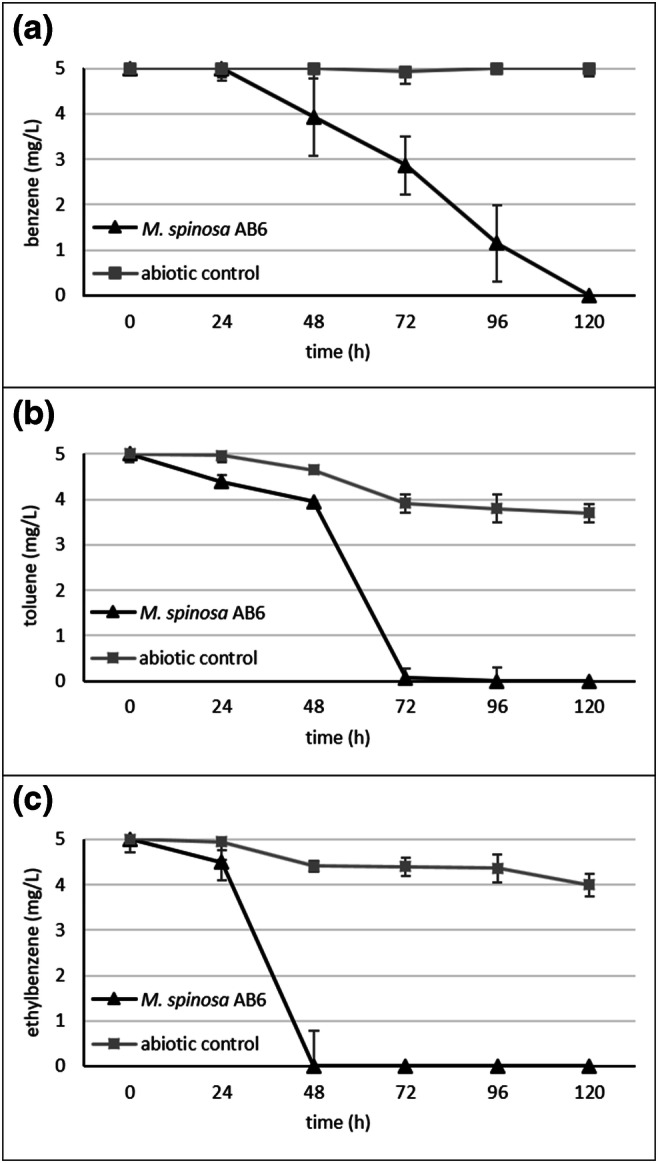
Aerobic degradation of **a** benzene, **b** toluene and **c** ethylbenzene by *Malikia spinosa* strain AB6. Concentrations were determined by GC–MS analysis as described in the main text. The averages of triplicate experiments ± standard errors of the means, indicated by error bars, are shown

**Fig. 5:**
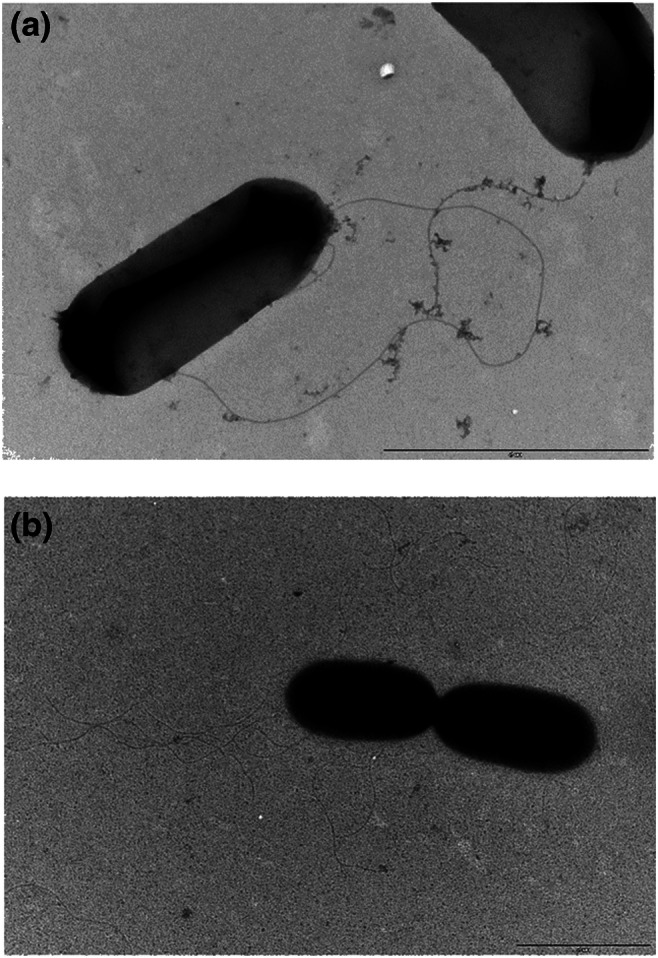
Transmission electron microscopic images of *Malikia spinosa* strain AB6 by using **a** negative staining and **b** shadow-casting technique. Bar, 2 μm

Bacterial strains were isolated from those enrichments that have been analysed by Illumina 16S rDNA amplicon sequencing. Not surprisingly, the diversity of the isolates was low. Altogether 22 strains were isolated, which represented only four genera (Table 1). Strains isolated from the aerobic benzene-degrading enrichment AB1 were members of the genera *Pseudomonas*, *Acidovorax* and *Malikia*. The *Malikia*-related isolates were identified as *Malikia spinosa* strains (strains AB2 and AB6), showing 99.7% 16S rDNA similarity to the type strain *M. spinosa* 83^T^. Moreover, both of these strains harboured subfamily I.2.C-type C23O gene. Sequence analysis of their C23O amplicons revealed that these were 100% identical with the sole C23O genotype found in the aerobic enrichments AB1 and AT2 (Fig. 3). For the analysis of aerobic BTEX degradation capability, the *M. spinosa* strain AB6 was chosen. As a result, it was observed that strain AB6 was able to degrade benzene, toluene and ethylbenzene (Fig. 4), while it was unable to degrade any isomer of xylene. According to the literature, this was the first time when a *Malikia spinosa* strain could be identified as an aromatic hydrocarbon–degrading bacterium. Besides, these results explained why members of the genus *Malikia* can be dominant bacteria of non-oxygen-limited, BTEX-contaminated environments. Transmission electron microscopic observations of strain AB6 revealed cell morphology and flagellation patterns similar to that of was reported in the case of the type strain 83^T^ (Spring et al. 2005) (Fig. 5). Strain AB5, which was identified as *Acidovorax delafieldii*, also harboured subfamily I.2.C-type C23O gene. This extradiol dioxygenase genotype was observed earlier by us in a microaerobic, BTEX-degrading enrichment culture (Benedek et al. 2018) (Fig. 3). The BTEX-degrading ability of strain AB5 was also investigated, and it turned out that it is able to degrade benzene only (result not shown). This ability of *Acidovorax* strains has already been suggested in oxygen-limited, BTEX-contaminated subsurface environments by different studies (Fahy et al. 2006; Aburto and Peimbert 2011).

**Table 1:** Identity of bacterial strains isolated from the enrichments

Strain No.	Closest relative (type strain)	length of 16S rDNA analysed (bp)	Similarity (%)	Subfamily I.2.C C23O gene
Aerobic benzene-degrading enrichment culture AB1				
AB1	*Pseudomonas arsenicoxydans* CECT 7543^T^	1437	99.4	–
AB2	*Malikia spinosa* ATCC 14606^T^	1432	99.7	+
AB3	*Pseudomonas arsenicoxydans* CECT 7543^T^	1437	99.4	–
AB4	*Pseudomonas veronii* DSM 11331^T^	1434	99.8	–
AB5	*Acidovorax delafieldii* DSM 64^T^	1444	99.9	+
AB6	*Malikia spinosa* ATCC 14606^T^	1428	99.9	+
Aerobic toluene-degrading enrichment culture AT2				
AT1	*Pseudomonas umsongensis* DSM 16611^T^	1436	99.9	–
AT2	*Pseudomonas veronii* DSM 11331^T^	1435	99.7	–
AT3	*Pseudomonas moorei* RW10^T^	1437	99.9	–
AT4	*Flavobacterium oncorhynchi* CCUG 59446^T^	1411	99.6	–
AT5	*Flavobacterium oncorhynchi* CCUG 59446^T^	1392	99.6	–
AT6	*Pseudomonas umsongensis* DSM 16611^T^	1435	99.9	–
Microaerobic benzene-containing enrichment culture MB1				
MB1	*Pseudomonas veronii* DSM 11331^T^	1437	99.8	–
MB2	*Pseudomonas veronii* DSM 11331^T^	1435	99.8	–
MB3	*Pseudomonas veronii* DSM 11331^T^	1436	99.8	–
MB4	*Pseudomonas veronii* DSM 11331^T^	1411	99.8	–
MB6	*Pseudomonas extremaustralis* 14-3^T^	1330	99.6	–
Microaerobic toluene-degrading enrichment culture MT3				
MT1	*Pseudomonas veronii* DSM 11331^T^	1438	99.8	–
MT2	*Pseudomonas veronii* DSM 11331^T^	1438	99.8	–
MT3	*Pseudomonas veronii* DSM 11331^T^	1439	99.8	–
MT4	*Pseudomonas veronii* DSM 11331^T^	1438	99.7	–
MT5	*Pseudomonas veronii* DSM 11331^T^	1451	99.8	–

Strains isolated from the aerobic toluene-degrading enrichment AT2 were members of the genera *Pseudomonas* and *Flavobacterium*. Unfortunately, none of the strains harboured subfamily I.2.C-type C23O gene. Since the toluene-degrading capability of *Pseudomonas* strains is well known, they were not investigated further. BTEX-degrading ability of strain AT4, identified as *Flavobacterium oncorhynchi*, was investigated, but no such ability was observed.

All of the strains isolated from the microaerobic enrichments belonged to the *Pseudomonas veronii*/*extremaustralis* lineage. Most importantly, none of them harboured subfamily I.2.C-type C23O gene and they were unable to degrade benzene. This is in accordance with our previous result that *P. veronii* isolates enriched under microaerobic conditions from the same groundwater well were also unable to degrade benzene, and were only able to degrade toluene and *m*- and *p*-xylene (Benedek et al. 2018). It is highly assumable that these *Pseudomonas veronii*/*extremaustralis*–related bacteria were responsible for toluene degradation in the microaerobic, toluene-degrading enrichment cultures. Although these strains did not harbour subfamily I.2.C-type C23O gene, this lineage of the genus *Pseudomonas* seems to be adapted to microaerobic environments. Earlier we have observed the dominance of these bacteria in microaerobic, crude oil/gasoline mixture–degrading enrichment cultures (Benedek et al. 2018; Révész et al. 2020), and it was reported that *P. extremaustralis* preferably degrades alkanes under microaerobic conditions (Tribelli et al. 2018).

### Whole-genome analysis of *Malikia spinosa* strain AB6 in the light of its aromatic hydrocarbon–degrading ability

**Fig. 6:**
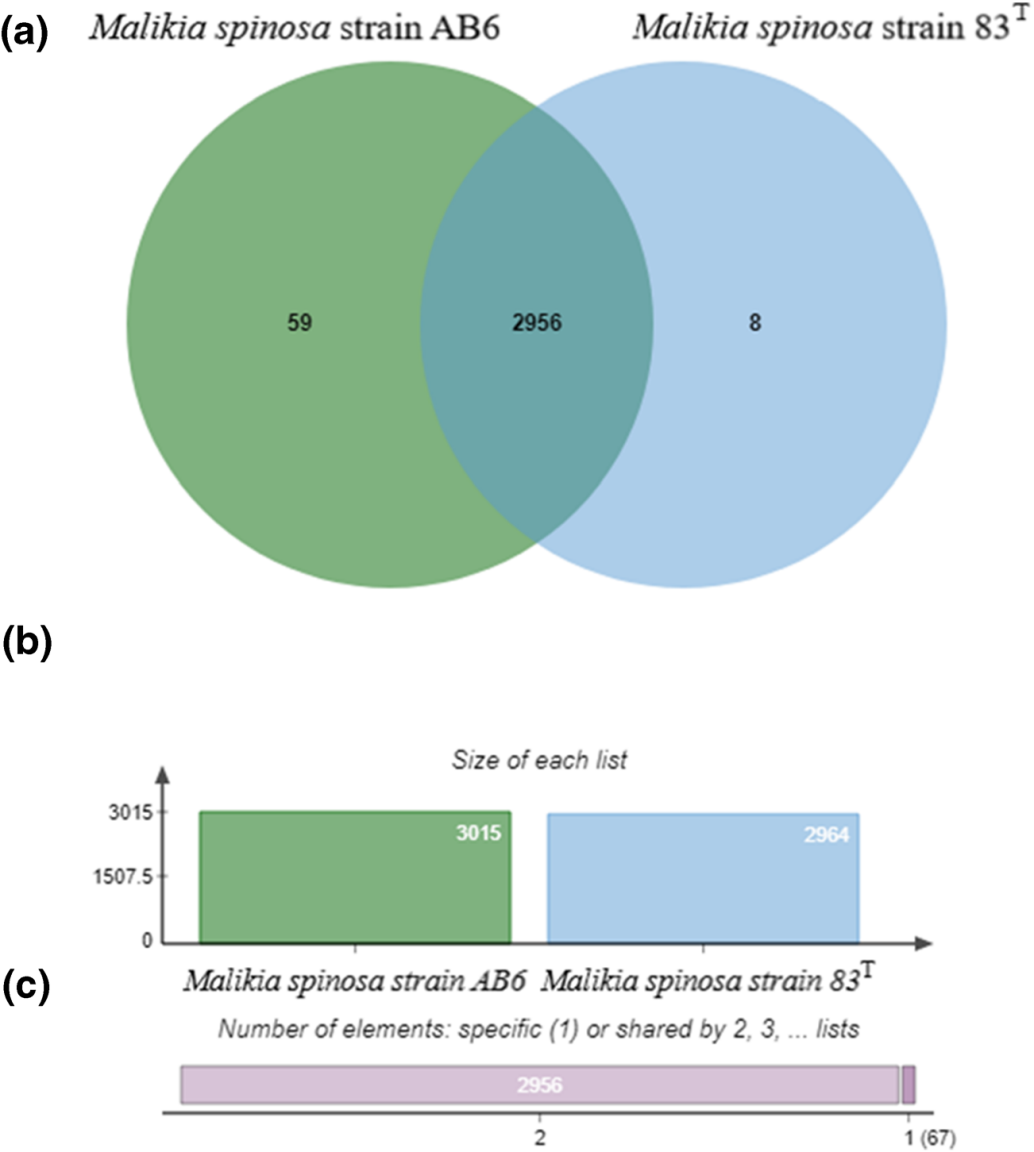
Comparative genomics analysis of *Malikia spinosa* strain AB6 and *Malikia spinosa* type strain 83^T^. **a** Venn diagram showing the distribution of shared gene families (orthologous clusters) among the two genomes. **b** Totals of orthologs in each genome that were used to generate the Venn diagram. **c** Number of shared (2) and specific (1) orthologs. The cluster number in each component is listed

The whole genome of *M. spinosa* strain AB6 was assembled into 143 contigs with 4,110,698 bps, 3868 total genes and 3614 protein coding genes (scaffold N50: 157 316; avg. coverage, 21.2; contamination, 0; completeness: complete). The G+C content of the genome is 65.2%. The draft genome sequence of strain AB6 has been deposited at the GenBank database under the WGS accession number VYSB00000000 (Bioproject: PRJNA572590; Biosample: SAMN12796314). Comparing the genome sequences of strain AB6 and the type strain *M. spinosa* 83^T^ (accession number PVLR00000000.1) revealed that strain AB6 has a ~ 300 Kbp larger genome than that of the type strain 83^T^. Although both dDDH and OrthoANI analyses showed that strains AB6 and 83^T^ belong to the same genomic species (79.4% and 97.7%, respectively), pan-genomic analysis based on the OrthoVenn2 tool showed significant differences between the two genomes (Fig. 6). The analysis identified 59 clusters of proteins, containing 148 proteins, which were coded only in the genome of strain AB6 and no orthologs or paralogs of these enzymes were coded in the genome of strain 83^T^. Moreover, a notable amount of these proteins could be linked to aromatic compound catabolic processes (data not shown). Annotation of the genome sequence of strain AB6 identified at least 48 genes affiliated with aromatic hydrocarbon degradation (according to SEED), while this number was only 28 in the case of strain 83^T^.

**Fig. 7:**
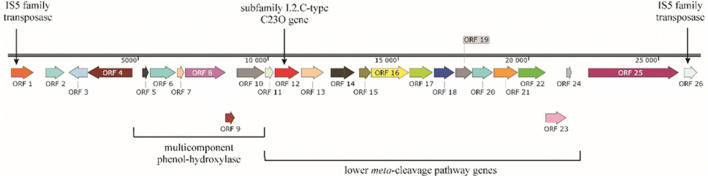
Schematic representation of the phenol-degradation cluster in the genome of *Malikia spinosa* AB6. Arrows indicate the orientation of the ORFs. Table S1 of Online Resource 2 contains the detailed description of the ORFs

Detailed analysis of the genome of strain AB6 revealed that the subfamily I.2.C-type C23O gene was part of a phenol degradation cluster, which coded enzymes of a multicomponent phenol hydroxylase system and a complete *meta*-cleavage pathway (Fig. 7). Most notably, this cluster was flanked by IS5 family transposases, which fact enables us to presume that this cluster was acquired by strain AB6 through a horizontal gene transfer (HGT) event. The origin of this gene cluster cannot be exactly determined due to the fact that the proteins coded by this cluster show considerably low amino acid sequence similarities to homologous proteins coded by known bacteria (Online Resource 2 Table S1). Since no other benzene/toluene mono- and/or dioxygenase was found in the genome, it is highly assumable that this phenol-degradation cluster is responsible for both benzene- and toluene-degrading abilities of strain AB6. A similar observation was made in the case of other Comamonadaceae family–related benzene and toluene degraders, e.g. *Alicycliphilus denitrificans* strains BC and K601^T^ (Oosterkamp et al. 2013). Hydroxylation of the benzene ring catalysed by phenol monooxygenases is a well-described process (Pérez-Pantoja et al. 2010), and their role in toluene degradation is also well documented (Cafaro et al. 2004; Martínez-Lavanchy et al. 2015).

Although ethylbenzene dioxygenase coding gene was not found in the genome of strain AB6, it was able to degrade ethylbenzene. Beside the pathway using the aromatic ring oxidation of ethylbenzene, the ethyl group oxidation catalysed by naphthalene dioxygenase is also feasible (Lee and Gibson 1996; Lee et al. 2019). Screening of the genome sequence for dioxygenases revealed the presence of genes, encoding the small and large subunits of a naphthalene dioxygenase, and were part of a naphthalene degradation gene cluster (i.e. a *nag* operon). This operon encoded salicylate-5-hydroxylase and gentisate 1,2-dioxygenase enzymes as well, which were reported earlier as the key naphthalene metabolic enzymes in the case of *Polaromonas naphthalenivorans* CJ2 and *Ralstonia* sp. U2 (Park et al. 2007; Pumphrey and Madsen 2007; Zhou et al. 2001). Thereby, it can be postulated that strain AB6 could degrade ethylbenzene due to the presence of the *nag* operon, and it probably has the ability to degrade naphthalene as well.

## Concluding remarks

Results of the present study shed light on the aromatic hydrocarbon–degrading ability of *Malikia spinosa* strains, thus providing evidence on our earlier presumption that this bacterium can act as a degrader in BTEX-contaminated environments, but mostly under strictly aerobic conditions (Táncsics et al. 2010; Benedek et al. 2018). This observation also revealed that bacteria, which encode subfamily I.2.C-type extradiol dioxygenase enzyme, will not be automatically able to degrade monoaromatic hydrocarbons under microaerobic conditions. Indeed, harbouring a subfamily I.2.C-type C23O gene is not the sole requirement of microaerobic aromatic hydrocarbon degradation, which was also shown by us earlier in the case of *Zoogloea oleivorans* strain Buc^T^ (Táncsics et al. 2020). Moreover, based on the whole-genome analysis of *Malikia spinosa* strain AB6, it can be suggested that subfamily I.2.C-type C23O genes easily spread among members of the Betaproteobacteriales through transposon-mediated HGT events.

## Electronic supplementary material

ESM 1 (PDF 436 kb)

ESM 2 (PDF 306 kb)
