# Supplementary material for: Effect of oxygen limitation on the enrichment of bacteria degrading either benzene or toluene and the identification of Malikia spinosa (Comamonadaceae) as prominent aerobic benzene-, toluene-, and ethylbenzene-degrading bacterium: enrichment, isolation and whole-genome analysis
Source: Environ Sci Pollut Res Int. 2020 May 30;27(25):31130–42. doi: 10.1007/s11356-020-09277-z (PMC7392937; doi:10.1007/s11356-020-09277-z)
Supplement: Supplementary file 1 — (PDF 436 kb) [file 11356_2020_9277_MOESM1_ESM.pdf]

Effect of oxygen-limitation on the enrichment of bacteria degrading either benzene or toluene and the identification of *Malikia spinosa* (*Comamonadaceae*) as prominent aerobic benzene-, toluene-, and ethylbenzene-degrading bacterium: enrichment, isolation and whole genome analysis.

Fruzsina Révész[1,2], Milán Farkas[1,2], Balázs Kriszt[1,2], Sándor Szoboszlay[2], Tibor Benedek[1,2], András Táncsics[1,2,*]

[1]Regional University Center of Excellence in Environmental Industry, Szent István University, Gödöllő, Hungary

[2]Department of Environmental Safety and Ecotoxicology, Szent István University, Gödöllő, Hungary

Journal: Environmental Science and Pollution Research

*Corresponding author: András Táncsics, Szent István University, Páter K. u. 1., 2100 Gödöllő, Hungary, tancsics.andras@fh.szie.hu, tel.: 06 28 522 000 #1611

Inoculum (biofilm)

Class level

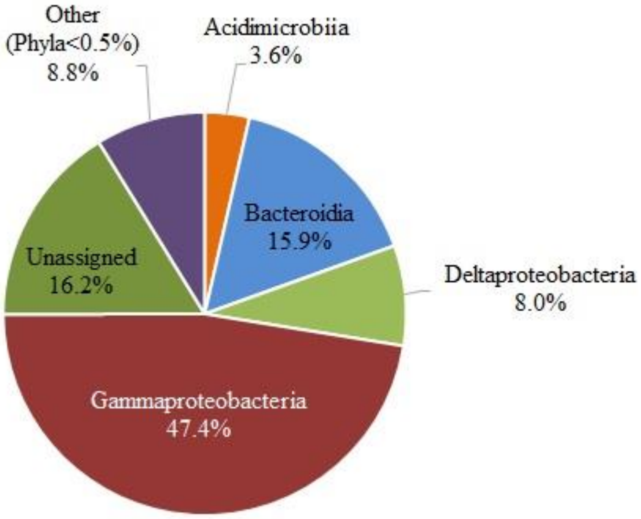

Genus level

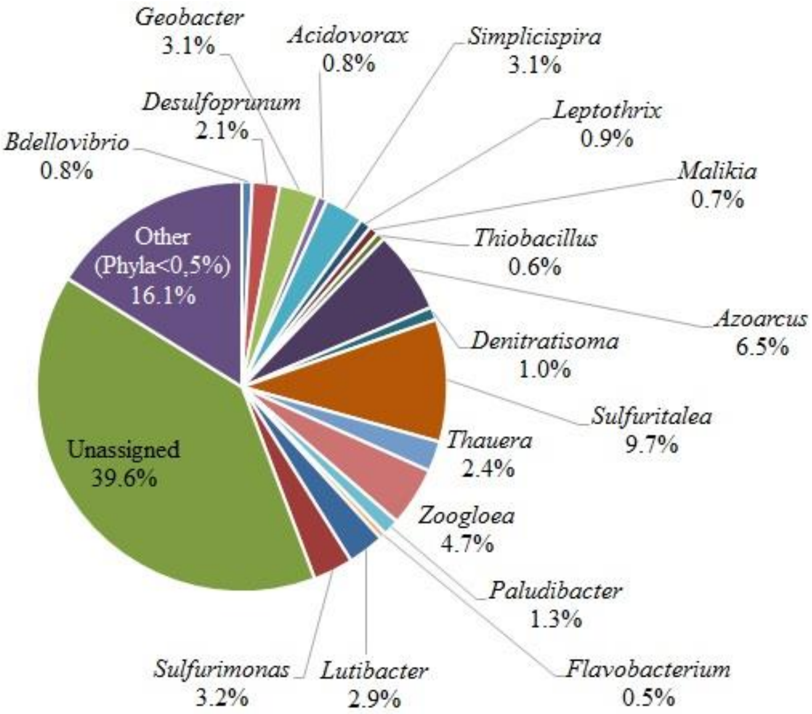

Figure S1: Bacterial community structure of the biofilm used as inoculant in the enrichments as revealed by Illumina paired-end 16S rDNA amplicon sequencing. All taxa contributing more than 0.5% abundance were depicted.

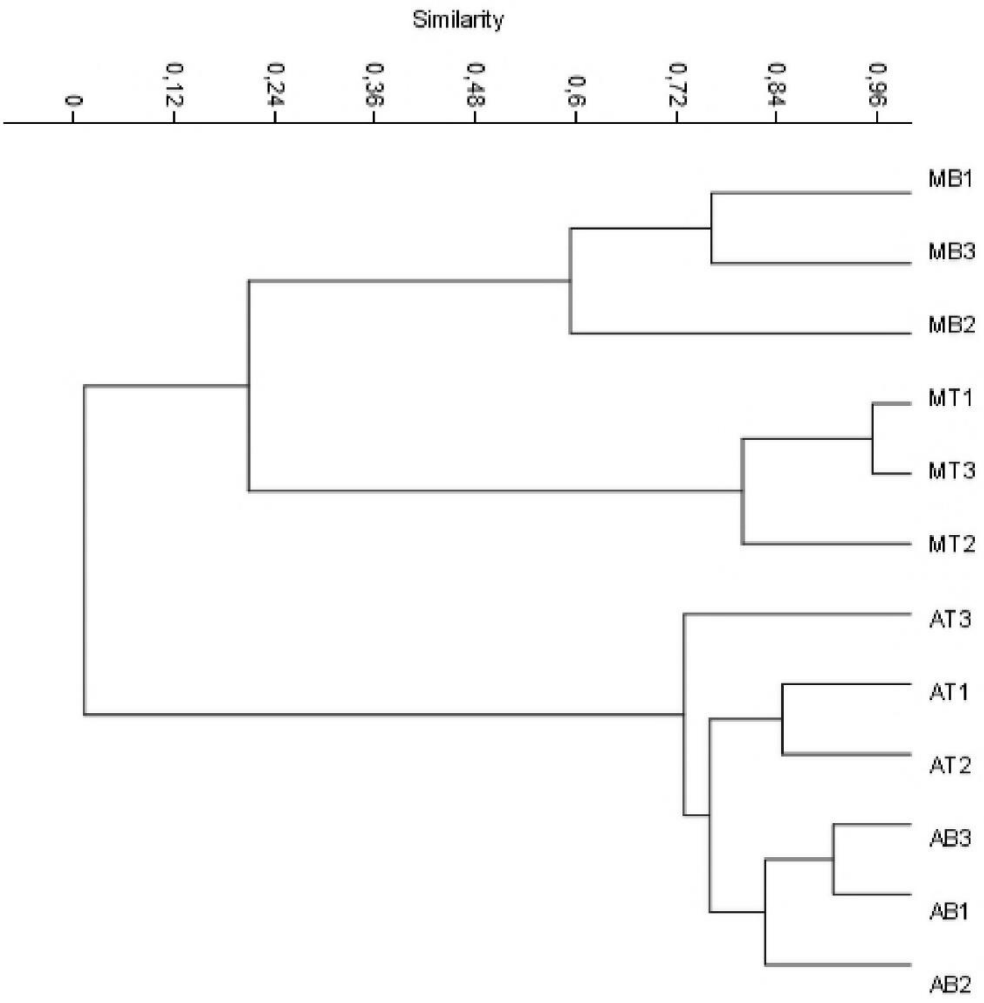

Figure S2: Cluster analysis of the 16S rDNA-based T-RFLP electropherograms of the triplicate enrichment cultures at the 5th week by Bray-Curtis algorithm. MB: microaerobic benzene-degrading enrichments; MT: microaerobic toluene-degrading enrichments; AB: aerobic benzene-degrading enrichments; AT: aerobic toluene-degrading enrichments.

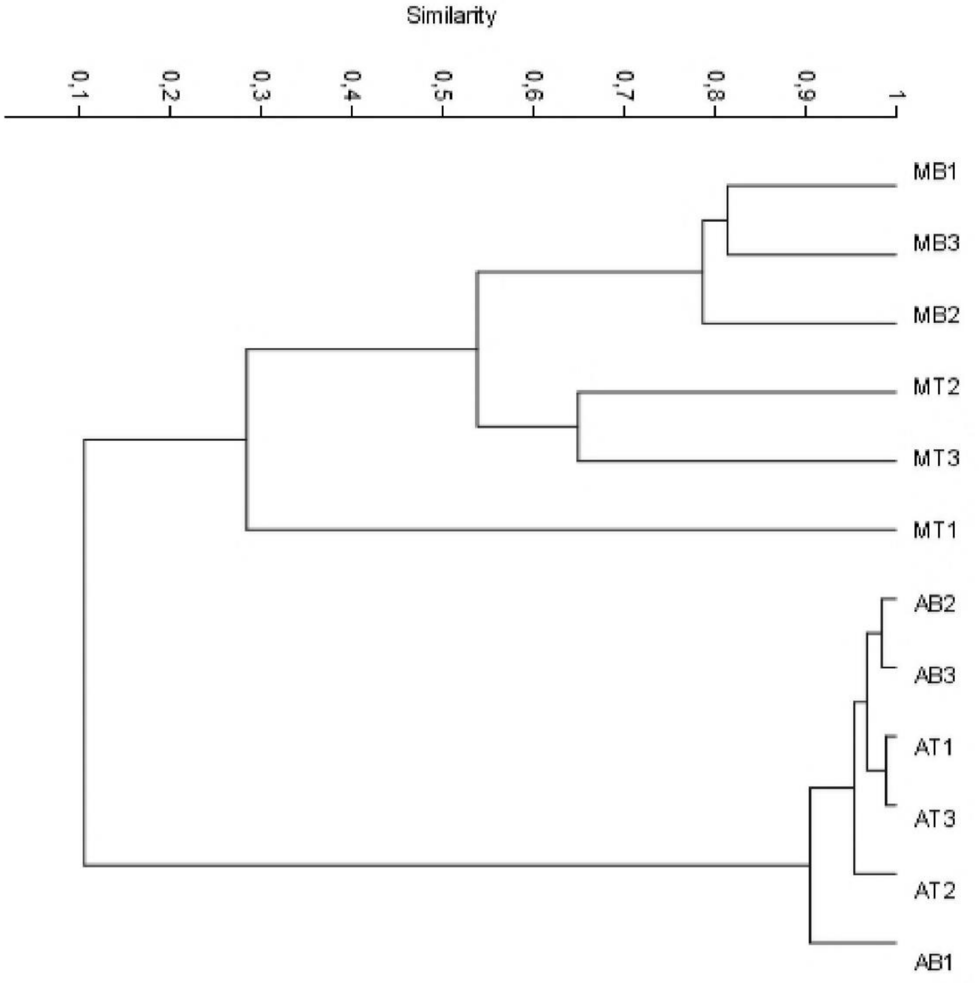

Figure S3: Cluster analysis of the subfamily I.2.C-type C23O gene-based T-RFLP electropherograms of the triplicate enrichment cultures at the 5th week by Bray-Curtis algorithm. MB: microaerobic benzene-degrading enrichments; MT: microaerobic toluene-degrading enrichments; AB: aerobic benzene-degrading enrichments; AT: aerobic toluene-degrading enrichments.
