# Supplementary material for: Effect of oxygen limitation on the enrichment of bacteria degrading either benzene or toluene and the identification of Malikia spinosa (Comamonadaceae) as prominent aerobic benzene-, toluene-, and ethylbenzene-degrading bacterium: enrichment, isolation and whole-genome analysis
Source: Environ Sci Pollut Res Int. 2020 May 30;27(25):31130–42. doi: 10.1007/s11356-020-09277-z (PMC7392937; doi:10.1007/s11356-020-09277-z)
Supplement: Supplementary file 2 — (PDF 306 kb) [file 11356_2020_9277_MOESM2_ESM.pdf]

Effect of oxygen-limitation on the enrichment of bacteria degrading either benzene or toluene and the identification of *Malikia spinosa* (*Comamonadaceae*) as prominent aerobic benzene-, toluene-, and ethylbenzene-degrading bacterium: enrichment, isolation and whole genome analysis.

Fruzsina Révész[1,2], Milán Farkas[1,2], Balázs Kriszt[1,2], Sándor Szoboszlay[2], Tibor Benedek[1,2], András Táncsics[1,2,*]

[1]Regional University Center of Excellence in Environmental Industry, Szent István University, Gödöllő, Hungary

[2]Department of Environmental Safety and Ecotoxicology, Szent István University, Gödöllő, Hungary

Journal: Environmental Science and Pollution Research

*Corresponding author: András Táncsics, Szent István University, Páter K. u. 1., 2100 Gödöllő, Hungary, tancsics.andras@fh.szie.hu, tel.: 06 28 522 000 #1611

Table S1: Description of the ORFs of the phenol-degradation gene cluster of *Malikia spinosa* strain AB6

ORF	Enzyme	Most similar enzyme coding organism	% similarity (aa)*	locus tag
1	IS5 family transposase	*Malikia spinosa* strain ATCC 14606[T]	94.7	F5985_01815
2	glutathione S-transferase	*Macromonas bipunctata* strain DSM 12705[T]	69.8	F5985_01810
3	FCD-domain containing protein	*Ralstonia pickettii* strain 12J	64.9	F5985_01805
4	sigma-54-dependent Fis family transcriptional regulator	*Verminephrobacter eiseniae* strain EF01-2	73.0	F5985_01800
5	phenol hydroxylase P0 protein	*Hydrogenophaga crassostreaea* strain LPB0072	59.3	F5985_01795
6	phenol hydroxylase P1 protein	*Hydrogenophaga* sp. strain T4	90.0	F5985_01790
7	phenol hydroxylase P2 protein	*Hydrogenophaga* sp. strain T4	88.8	F5985_01785
8	phenol hydroxylase P3 protein	*Hydrogenophaga* sp. strain T4	92.7	F5985_01780
9	phenol hydroxylase P4 protein	*Hydrogenophaga* sp. strain T4	81.4	F5985_01775
10	2Fe-2S iron-sulfur cluster binding domain-containing protein	*Pseudoxanthomonas spadix* strain BD-a59	87.3	F5985_01770
11	ferredoxin	*Zoogloea oleivorans* Buc[T]	85.1	F5985_01765
12	catechol 2,3-dioxygenase	*Pseudoxanthomonas spadix* strain BD-a59	91.7	F5985_01760
13	transporter	*Alicycliphilus denitrificans* strain BQ1	67.6	F5985_01755
14	LysR family transcriptional regulator	*Thauera* sp. D20	79.3	F5985_01750
15	heme-binding protein	*Pseudoxanthomonas spadix* strain BD-a59	83.7	F5985_01745
16	2-hydroxymuconic semialdehyde dehydrogenase	*Zoogloea ramigera* strain NBRC 15342[T]	93.0	F5985_01740
17	2-hydroxymuconate semialdehyde hydrolase	*Herminiimonas* sp. strain CN.	86.5	F5985_01735
18	2-hydroxypent-2,4-dienoate hydratase	*Hydrogenophaga* sp. strain T4	91.2	F5985_01730
19	hypothetical protein	*Hydrogenophaga* sp. strain T4	70.7	F5985_01725

20	SDR family oxidoreductase	*Polaromonas* sp. AER18D-145	79.5	F5985_01720
21	acetaldehyde dehydrogenase	*Ralstonia* sp. NFACC01	90.6	F5985_01715
22	4-hydroxy-2-oxovalerate aldolase	*Pseudoxanthomonas spadix* strain BD-a59	90.5	F5985_01710
23	2-oxo-3-hexenedioate decarboxylase	*Polaromonas* sp. AER18D-145	89.7	F5985_01705
24	4-oxalocrotonate tautomerase family protein	*Simplicispira metamorpha* strain DSM 1837^{T}	93.7	F5985_01700
25	pyruvate carboxylase	*Malikia spinosa* strain ATCC 14606^{T}	94.6	F5985_01690
26	IS5 family transposase	*Malikia spinosa* strain ATCC 14606^{T}	90.0	F5985_01685

*aa: amino acid
